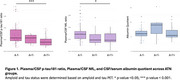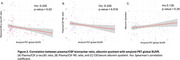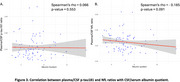# Impaired CSF Clearance Contributes to the Retention of Alzheimer's Biomarkers Independent of Blood‐Brain Barrier Dysfunction

**DOI:** 10.1002/alz70862_110073

**Published:** 2025-12-23

**Authors:** Tevy Chan, Seyyed Ali Hosseini, Arthur C. Macedo, Lydia Trudel, Marina P Gonçalves, Nesrine Rahmouni, Brandon J Hall, Joseph Therriault, Kely Monica Quispialaya Socualaya, Stijn Servaes, Gleb Bezgin, Yansheng Zheng, Yi‐Ting Wang, Etienne Aumont, Jaime Fernandez Arias, Ana Paula Bernardes Real, Wan Lu Jia, Robert Hopewell, Chris Hsiao, Paolo Vitali, Tharick A Pascoal, Pedro Rosa‐Neto

**Affiliations:** ^1^ Montreal Neurological Institute, Montreal, QC Canada; ^2^ McGill University, Montreal, QC Canada; ^3^ Translational Neuroimaging Laboratory, The McGill University Research Centre for Studies in Aging, Montréal, QC Canada; ^4^ The McGill University Research Centre for Studies in Aging, Montreal, QC Canada; ^5^ Université du Québec à Montréal, Montréal, QC Canada; ^6^ University of Pittsburgh, Pittsburgh, PA USA; ^7^ Departments of Psychiatry and Neurology, University of Pittsburgh School of Medicine, Pittsburgh, PA USA; ^8^ McConnell Brain Imaging Centre, Montreal Neurological Institute, McGill University, Montreal, QC Canada

## Abstract

**Background:**

Ratio between plasma and cerebrospinal fluid (CSF) biomarkers might inform about the peptide clearance from the central nervous system (CNS) to the peripheral body compartments. However, whether this clearance is linked to blood‐brain barrier (BBB) alterations in Alzheimer's disease (AD) remains unclear. In this study, we examined the ratio between plasma and CSF neurofilament light chain (NfL) and *p*‐tau181 and its relationship with BBB permeability in individuals across the AD spectrum.

**Method:**

We analyzed data of 102 participants (median age 66 years, 54% female) from the Translational Biomarkers in Aging and Dementia (TRIAD) cohort. Plasma and CSF levels of NfL and *p*‐tau181 were measured using Lumipulse G1200 (Fujirebio). The CSF/serum albumin quotient, a marker of BBB integrity, was calculated, where higher values indicate increased permeability. Mann‐Whitney U test compared the biomarker ratios among ATN groups. Spearman’s correlation examined the association between the plasma/CSF biomarker ratios and amyloid PET (^18^F‐NAV4694) global standardized uptake value ratio (SUVR), and between plasma/CSF biomarker ratios and the albumin quotient.

**Result:**

Plasma *p*‐tau181/CSF *p*‐tau181 ratio and plasma NfL/CSF NfL ratio were decreased in A+T+ individuals (Figure 1), suggesting impaired CSF clearance in AD. No significant differences in albumin quotient values were observed among ATN groups. Moreoever, increased amyloid burden on PET correlated with reduced plasma/CSF *p*‐tau181 and NfL ratios (Figure 2), further supporting a link between amyloid pathology and impaired clearance mechanisms. Finally, no significant correlation was found between plasma/CSF *p*‐tau181 and NfL ratios and albumin quotient (Figure 3), suggesting that CSF clearance deficit is independent of BBB integrity.

**Conclusion:**

Our results support the concept that impaired CSF clearance contributes to the accumulation of AD biomarkers in the CNS. Moreover, the impaired CSF clearance does not seem to be associated with BBB dysfunction. These results highlight CSF clearance as a potential therapeutic target for AD, emphasizing the need to explore mechanisms that enhance peptide drainage from the CNS.